# Radiomics Profiling Identifies the Incremental Value of MRI Features beyond Key Molecular Biomarkers for the Risk Stratification of High-Grade Gliomas

**DOI:** 10.1155/2022/8952357

**Published:** 2022-03-23

**Authors:** Guoqiang Yang, Yongjian Sha, Xiaochun Wang, Yan Tan, Hui Zhang

**Affiliations:** ^1^Department of Radiology, First Clinical Medical College, Shanxi Medical University, Taiyuan 030001, Shanxi, China; ^2^College of Medical Imaging, Shanxi Medical University, Taiyuan 030001, Shanxi, China

## Abstract

**Objective:**

To identify the incremental value of magnetic resonance imaging (MRI) features beyond key molecular biomarkers for the risk stratification of high-grade gliomas (HGGs).

**Methods:**

A total of 241 patients with preoperative magnetic resonance (MR) images and clinical and genetic data were retrospectively collected from our institution and The Cancer Genome Atlas/The Cancer Imaging Archive (TCGA/TCIA) dataset. Radiomic features (*n* = 1702) were extracted from both postcontrast T1-weighted (CE-T1) and T2-weighted fluid attenuation inversion recovery (T2FLAIR) MR images. The least absolute shrinkage and selection operator (LASSO) method was used to select effective features. A multivariate Cox proportional risk regression model was established to explore the prognostic value of clinical features, molecular biomarkers, and radiomic features. Kaplan–Meier survival analysis and the log-rank test were used to evaluate the prognostic model, and a stratified analysis was conducted to demonstrate the incremental value of the radiomics signature. A nomogram was developed to predict the 1-year, 2-year, and 3-year overall survival (OS) probabilities of the patients with HGGs.

**Results:**

The radiomics signature provided significant prognostic value for the risk stratification of patients with HGGs. The combined model integrating the radiomics signature with clinical data (age) and O^6^-methylguanine-DNA methyltransferase (MGMT) promoter methylation status had the best prognostic value, with C-index values of 0.752 and 0.792 in the training set and external validation set, respectively. Stratified Kaplan–Meier survival analysis showed that the radiomics signature could identify the risk subgroups in different clinical and molecular subgroups.

**Conclusion:**

This radiomics signature can be used for the risk stratification of patients with HGGs and has incremental value beyond key molecular biomarkers, providing a preoperative basis for individualized diagnosis and treatment decision-making.

## 1. Introduction

High-grade gliomas (HGGs) are the most common primary adult brain malignancy of the central nervous system (CNS), and they comprise 2 World Health Organization (WHO) grades (grade III-IV gliomas) [1, 2]. The prognosis of patients with HGGs is poor despite the best available therapies. Patients with higher pathological grades experience a worse prognosis; for those with grade III gliomas, the five-year survival rates range from 27.3% to 52.2%, and for those with grade IV gliomas, the five-year survival rate is just 5% [3]. However, some HGGs with the same pathological grade have significant differences in curative efficacy and prognosis, and this is closely related to tumour genotyping. Tumours with isocitrate dehydrogenase (IDH) mutations are associated with a better survival outcome than those with wild-type IDH genes, independent of pathological grade [4, 5], and patients with grade III gliomas with wild-type IDH may even have a worse prognosis than those with grade IV gliomas with IDH mutations [6]. IDH has important diagnostic and prognostic value, making it a classification indicator in the WHO diagnostic criteria for gliomas [7]. In addition, MGMT as a key DNA repair protein has assisted chemotherapy decision-making and its methylation status is a prognostic predictor. HGGs with methylated MGMT exhibited good responses to temozolomide (TMZ) chemotherapy and are associated with improved survival outcomes after treatment [8, 9]. At present, IDH and MGMT are the most important molecular biomarkers for the prognostic evaluation of patients with HGGs [10].

Parallel to developments in the molecular classification of HGGs, magnetic resonance imaging (MRI), as a powerful noninvasive diagnostic imaging tool, provides the potential opportunity for preoperative risk evaluations in patients with HGGs. Radiomics has recently emerged as a promising field of research based on the hypothesis that quantitative analysis of medical images can capture additional information that helps infer phenotypes and gene-protein signatures and provide prognostic information [11, 12]. More recently, radiomics has been used to identify brain abscess from cystic gliomas [13], predict the molecular subtypes of HGGs (such as IDH [14, 15] and MGMT [16] status), assess the antiangiogenic treatment response of recurrent glioblastomas (GBMs) [17], and stratify the risk of patients with GBMs [18]. Previous radiomics analysis of GBMs has shown that the radiomics signature holds better prognostic value than clinical and radiological risk models in predicting survival [18]. The latest research further supports that the radiomics signature improves the stratification of patients with GBMs beyond the presence of MGMT molecular biomarkers [19]. However, the prognostic value of the radiomics signature has been demonstrated only in patients with GBMs, and those with grade III gliomas were not involved. Moreover, the key molecular biomarkers (IDH and MGMT) were not compared or integrated into the radiomics analysis. The incremental prognostic value of the radiomics signature needs further investigation through a comprehensive and stratified risk analysis.

In this study, 241 patients with HGGs from our institution and the TCGA/TCIA dataset were retrospectively investigated. We explored the possibility of using radiomic features extracted from preoperative MR images to stratify the risk of patients with HGGs and investigated the incremental prognostic value of the radiomics signature beyond key molecular biomarkers and clinical characteristics. Furthermore, a radiomics nomogram that incorporated both clinical and genetic factors and the radiomics signature was developed to predict the overall survival (OS) probability of individual patients with HGGs.

## 2. Materials and Methods

### 2.1. Patients

**Figure 1:**
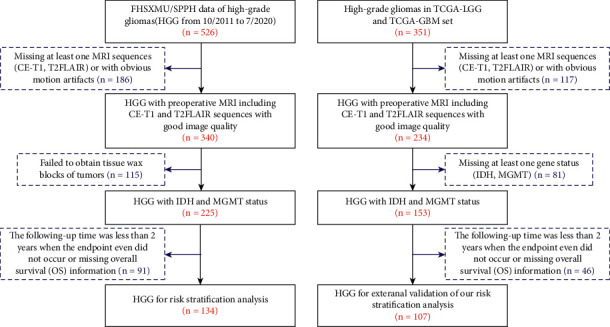
Flowchart of the patients with HGG included and excluded for the risk stratification analysis.

This retrospective study included patients with histologically confirmed WHO grade III-IV gliomas from our institutions, including the First Hospital of Shanxi Medical University (FHSXMU) and Shanxi Provincial People's Hospital (SPPH), and the TCGA/TCIA project. This retrospective study at our institution was conducted following approval by the Shanxi Medical University institutional review board. Under TCGA/TCIA data-use agreements, as the patients had been previously deidentified and their relevant information was available for public download, analysis of this cohort was exempt from institutional review board approval. All 526 patients with pathologically confirmed HGGs in our institution during the period from 10/2011 to 7/2020 were enrolled and screened. A total of 351 HGGs from two subdatasets of the TCGA/TCIA project were collected and screened including the TCGA-LGG and TCGA-GBM datasets. All the patients identified in this study met the following criteria: (i) pathologically confirmed grade III-IV gliomas according to current WHO criteria, (ii) available preoperative MRI data consisting of CE-T1 and T2FLAIR images, (iii) confirmed IDH and MGMT statuses, and (iv) a follow-up time longer than 2 years or an endpoint event (met the requirements for overall survival analysis). Overall survival was calculated from the time of the postoperative pathological diagnosis until death or the last follow-up. Ultimately, 241 patients who met the inclusion criteria (134 from FHSXMU/SPPH and 107 from the TCGA/TCIA dataset) were enrolled in the risk stratification study. A flowchart of the patients included and the reasons for exclusion from the risk stratification analysis are shown in Figure 1.

In addition, preoperative MRI was performed at our institution with a 3.0T scanner (Signa HDxt, GE Healthcare, USA) using an 8-channel array coil. The acquisition protocol included CE-T1 (repetition time/echo time, 195 ms/4.76 ms; field of view, 240 mm; thickness/slice interval, 5.0 mm/1.5 mm; and matrix, 256 × 256) and T2FLAIR (repetition time/echo time, 8000 ms/95 ms; field of view, 240 mm; thickness/slice interval, 5.0 mm/1.5 mm; and matrix, 256 × 256) images. The CE-T1 was performed after the injection of 0.1 mmol/kg gadolinium chelate contrast medium.

### 2.2. IDH Genotyping and MGMT Methylation Testing

For patients in the TCGA/TCIA cohort, IDH1/2 mutation and MGMT methylation data were downloaded from TCGA and cBioPortal for Cancer Genomics (https://www.cbioportal.org/study.do?cancer_study_%20id=lgggbm_tcga_pub). For patients at our institution, IDH1/2 mutation status was determined by Sanger sequencing. After dewaxing, each tumour specimen was histologically investigated by microdissection to guarantee a tumour cell content of at least 80%, while DNA was extracted using the Simlex OUP® FFPE DNA extraction kit (TIB, China) and quantified by spectrophotometry using a NanoDrop 2000 (Thermo Fisher Scientific, USA). Genes were amplified using PCR ABI9700 Life Technologies (Thermo Fisher Scientific, USA). Gene-specific primers (Primer-blast, NCBI) for IDH1 were F: 5′CGGTCTTCAGAGAAGCCATT3′ and R: 5′GCAAAATCACATTATTGCCAA3′ and those for IDH2 were F: 5′CAAGAGGATGGCTAGGCGAG3′ and R: 5′CAAGCTGAAGAAGATGTGGAAAAG3′. Sanger sequencing was performed by ABI3500 Life Technologies (Thermo Fisher Scientific, USA). As described in our previous study [20], MGMT methylation was evaluated using pyrosequencing analysis [21]. Bisulphite modification of the extracted DNA was performed using the BisulFlash™ DNA modification kit (Epigentek, USA). PCR amplification was accomplished with a DRR006 kit (Takara, Japan) using a 40 *μ*l reaction volume. The PCR conditions were as follows: 94°C for 2 min; 50 cycles at 94°C for 20 s, 55°C for 20 s, 72°C for 20 s, and 72°C for 5 min. A 25 *μ*l volume of the PCR product was subjected to pyrosequencing on a PyroMark Q96 (Qiagen, Germany). Pyrosequencing yielded data for 10 CpG sites within the MGMT promoter, and the percentage methylation obtained for each CpG was averaged across the 10 CpGs in PCRs. Tumours were considered to be methylated if the average methylation was ≥8%, and unmethylated cases had average methylation <8% [22].

### 2.3. Tumour ROI Segmentation

**Figure 2:**
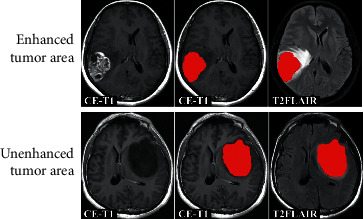
Delineation details of the ROI of enhanced and unenhanced tumours.

The T2FLAIR MR images were coregistered to the corresponding CE-T1 MR images using affine transformation through FSL software (FMRIB software library; FSL, https://fsl.fmrib.ox.ac.uk/fsl/fslwiki/FSL). To ensure the region of interest (ROI) delineation accuracy, manual segmentation was performed in a blinded fashion by 2 radiologists with 10 and 15 years of work experience. The final ROI was determined as the overlapping area of the segmentation results from the two radiologists and was validated by a senior radiologist with 20 years of experience. For the ROI of the enhanced tumour, the enhanced rim was the border of the tumour area, and the ROI of the tumour was delineated on CE-T1 MR images and then transferred to T2FLAIR MR images. For the ROI of unenhanced tumours, the intensity of the tumour was lower than that of peritumoral edema on CE-T1 MR images, and the ROI of the tumour was delineated on CE-T1 MR images and then transferred to T2FLAIR MR images. If the border of the tumour area was not very clear both on either CE-T1 or T2FLAIR MR images, we combined DWI and ADC MR images (if available) to define the borders. Tumour intensity was higher than peritumoral edema intensity on DWI MR images and lower than peritumoral edema intensity on ADC MR images. An ROI segmentation example for enhanced tumour and unenhanced tumour was shown in Figure 2 to highlight the segmentation details. To evaluate the stability and reproducibility of radiomic features, interrater analysis was carried out based on the segmentation results by the former two radiologists, and the interclass correlation coefficient (ICC) was used as a measure of the measurement index.

### 2.4. MRI Radiomic Feature Extraction

To generate well-defined input for the radiomic feature extraction, image resampling and image intensity normalization were conducted on CE-T1 and T2FLAIR MR images for all cases in both our institution and the TCGA/TCIA dataset using an in-house MATLAB (The MathWorks, Natick, MA) process. A total of 1702 radiomic features were extracted from the tumour ROI of well-defined CE-T1 and T2FLAIR MR images. Each MR sequence contains 851 features including 18 first-order features, 14 shape features, 75 texture features (consisting of 24 grey-level co-occurrence matrix (GLCM) features, 14 grey-level dependence matrix (GLDM) features, 16 grey-level run length matrix (GLRLM) features, 16 grey-level size zone matrix (GLSZM) features, and 5 neighbourhood grey-tone dependency matrix (NGTDM) features), and 744 wavelet features derived from first-order and texture features based on the wavelet filter MR image. Radiomic feature extraction was carried out using the feature extraction function of the open source software FAE (https://github.com/salan668/FAE), which is based on the PyRadiomics package (https://github.com/Radiomics/pyradiomics).

### 2.5. Statistical Analysis

The FHSXMU/SPPH data from our institution were used as the training set to build the prognostic model, and the TCGA/TCIA data were used as an external validation set to further evaluate the prognostic model. For continuous variables, Student's *t*-test or the Mann–Whitney *U* test was used to assess the statistical significance of differences. For categorical variables, Pearson's chi-square or Fisher's exact test was used. We performed the statistical analysis with SPSS, version 23.0 (SPSS Inc., Chicago, IL, USA) and R software, version 3.4.1 (https://www.R-project.org). A two-sided *p* value less than 0.05 was considered statistically significant.

To verify the stability and robustness of the radiomic features, features with an ICC lower than 0.8 were removed for further analysis. Then, the remaining stable features were normalized by a z-score transformation and screened as follows: First, a univariate Cox regression analysis was used to eliminate nonsignificant features with *p* values >0.05. Subsequently, the least absolute shrinkage and selection operator (LASSO) in the Cox proportional hazards regression model was used to select the optimal features. The tuning parameter *λ* as the global regularization parameter was identified via 10-fold cross-validation to shrink the coefficients of useless features to zero. Ultimately, the selected features were integrated into a radiomics signature, and the radiomics score (Rad-score) was also calculated for each patient via a linear combination of the final selected features with their respective weight coefficients. The prognostic predictive performance was assessed on the training set and validated on the independent external validation set using the C-index. Kaplan–Meier survival analysis and the log rank test were used to evaluate the performance of the radiomics signature. Patients in the training and validation sets were classified into low-risk and high-risk groups according to a fixed cut-off value. In addition, a stratified analysis was conducted in different clinical subgroups and molecular subgroups to demonstrate the prognostic value of the radiomics signature on the training and validation sets. The above process was realized by using the survival package and glmnet package of R software.

The univariate Cox model was used to assess the association between each clinical risk factor and OS. A multivariate Cox model with stepwise forward selection was performed to construct a clinic-genetic model using the minimum Akaike information criterion (AIC) as a model selection criterion. The C-index and net reclassification index (NRI) were used to assess the incremental value of the radiomics signature to clinic-genetic risk factors. Finally, a radiomics nomogram was constructed by incorporating the radiomics signature and independent clinic-genetic risk factors into the multivariate Cox model. The calibration curve was used to assess the agreement between the predicted survival probabilities and the actual survival. Decision curve analysis was performed to compare the clinical usefulness of the radiomics model, clinic-genetic model, and combined model.

## 3. Results

### 3.1. Patient Characteristics

In this study, 134 patients from our institution (FHSXMU/SPPH) and 107 patients from the TCGA/TCIA dataset were included. The characteristics of the patients are shown in Table 1. There were no significant differences between the training set and the external validation set in terms of age, sex, MGMT promoter methylation status, and OS (*p* = 0.212–0.986). However, there were significant differences between the two datasets in pathological grade and IDH genotype (*p* = 0.008 and *p* = 0.006, respectively). These differences should be due to the uneven distribution of the data in the training set and external validation set. GBM accounted for the majority (66.4%) of the HGGs in the TCGA/TCIA dataset, while GBM accounted for 49.3% of the HGGs in our dataset.

### 3.2. MRI Features and Radiomics Signature Construction

**Figure 3:**
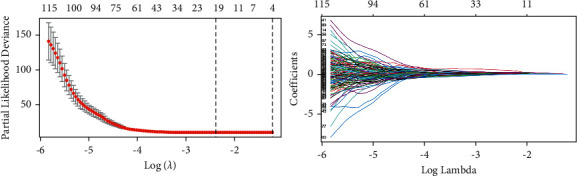
Dimension reduction of radiomic features in the LASSO Cox model.

**Figure 4:**
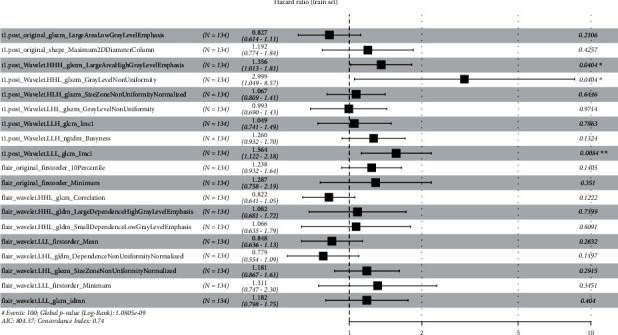
HR values with 95% CI and *p* values of each selected feature of the radiomics model on the forest map in the training set. Postcontrast axial T1-weighted (CE-T1) = t1.post; T2-weighted fluid attenuation inversion recovery (T2FLAIR) = flair.

Univariate Cox regression yielded 348 radiomic features for CE-T1 MR images and 54 radiomic features for T2FLAIR MR images. To select the best radiomic features and address the issue of overfitting, a LASSO Cox proportional risk regression model was adopted. Finally, at the minimum *λ* value, 19 MRI radiomic features (9 features from CE-T1, 10 features from T2FLAIR) with nonzero coefficients were retained (Figure 3), and the radiomics risk prediction model was established (Figure 4).

The constructed radiomics signature is as follows:

Signaturet1.post + flair = −0.19006*∗*t1.post_original_glszm_LargeAreaLowGrayLevelEmphasis+0.17586 *∗* t1.post _original_shape_Maximum2DDiameterColumn +0.30471 *∗* t1.post_wavelet.HHH_glszm_LargeAreaHighGrayLevelEmphasis +1.09819 *∗* t1.post_wavelet.HHL_glszm_GrayLevelNonUniformity +0.06513*∗* t1.post_wavelet.HLH_glszm_SizeZoneNonUniformityNormalized −0.00665 *∗* t1.post_wavelet.LHL_glszm_GrayLevelNonUniformity +0.04822*∗* t1.post_wavelet.LLH_glcm_Imc1 +0.23107*∗* t1.post_wavelet.LLH_ngtdm_Busyness +0.44740478 *∗* t1.post_wavelet.LLL_glcm_Imc1 +0.21320 *∗*flair_original_firstorder_10Percentile +0.25211*∗* flair_original_firstorder_Minimum -0.19597*∗*flair_wavelet.HHH_glcm_Correlation + 0.07843 *∗* flair_wavelet.HHL_gldm_LargeDependenceHighGrayLevelEmphasis +0.06384*∗* flair_wavelet.HHL_gldm_SmallDependenceLowGrayLevelEmphasis −0.16457*∗* flair_wavelet.HLL_firstorder_Mean −0.25002*∗* flair_wavelet.LHL_gldm_DependenceNonUniformity Normalized +0.16635*∗* flair_wavelet.LHL_glszm_SizeZoneNonUniformityNormalized +0.27067 *∗* flair_wavelet.LLL_firstorder_Minimum +0.16745*∗* flair_wavelet.LLL_glcm_Idmn

### 3.3. Evaluation of the Prognostic Performance of the Radiomics Model

**Figure 5:**
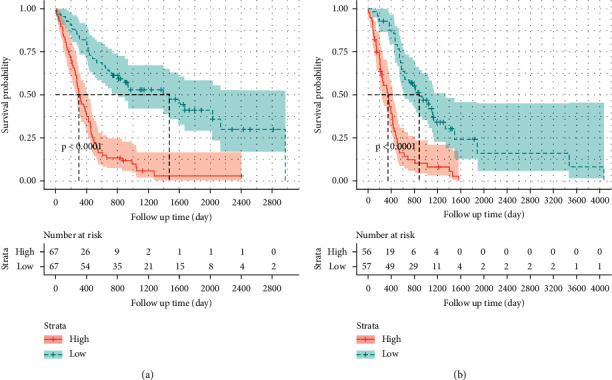
Kaplan–Meier curves of the radiomics model in the training set (a) and validation set (b). The banded curve represents the 95% CI at each point on the survival curve.

The prognostic predictive performance of the radiomics model, as evaluated by the C-index, was 0.745 on the training set and 0.750 on the independent external validation set. The radiomics risk score of each patient was calculated according to the radiomics signature, and the patients were divided into a high-risk group and a low-risk group based on a cut-off value (median = 1.071). Kaplan–Meier survival analysis, combined with the log-rank test, showed that the radiomics model achieved significant prognostic value in stratifying the high-risk and low-risk groups (*p* < 0.0001 for both the training set and the independent external validation set) (Figure 5).

### 3.4. Establishment of a Clinical-Genetic Model and Comparison with the Radiomics Model

**Figure 6:**
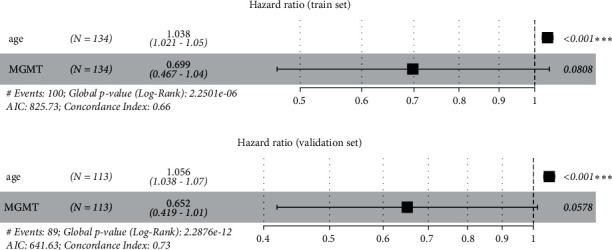
HR values with 95% CIs and *p* values for the clinical-genetic model in the training set and external validation set. The C-index is shown on the forest map.

A clinical-genetic model was constructed by using the multivariate Cox regression model with the minimum AIC as the model selection criterion. The clinical and genetic variables finally selected are shown in Figure 6, and the C-indexes of the clinical-genetic model were 0.660 and 0.730 with the minimum AIC in the training set and external validation set, respectively. The NRI was used to compare the 3-year survival prediction gains of the radiomics model and the clinical-genetic model. The patients were divided into high-risk and low-risk groups by the median (0.792) of the predicted values of the radiomics model. The NRI was 0.127 (95% CI: −0.005–0.398, *p* < 0.001) in the radiomics model versus the clinical-genetic model.

### 3.5. Stratified Kaplan–Meier Survival Analysis of the Radiomics Model

**Figure 7:**
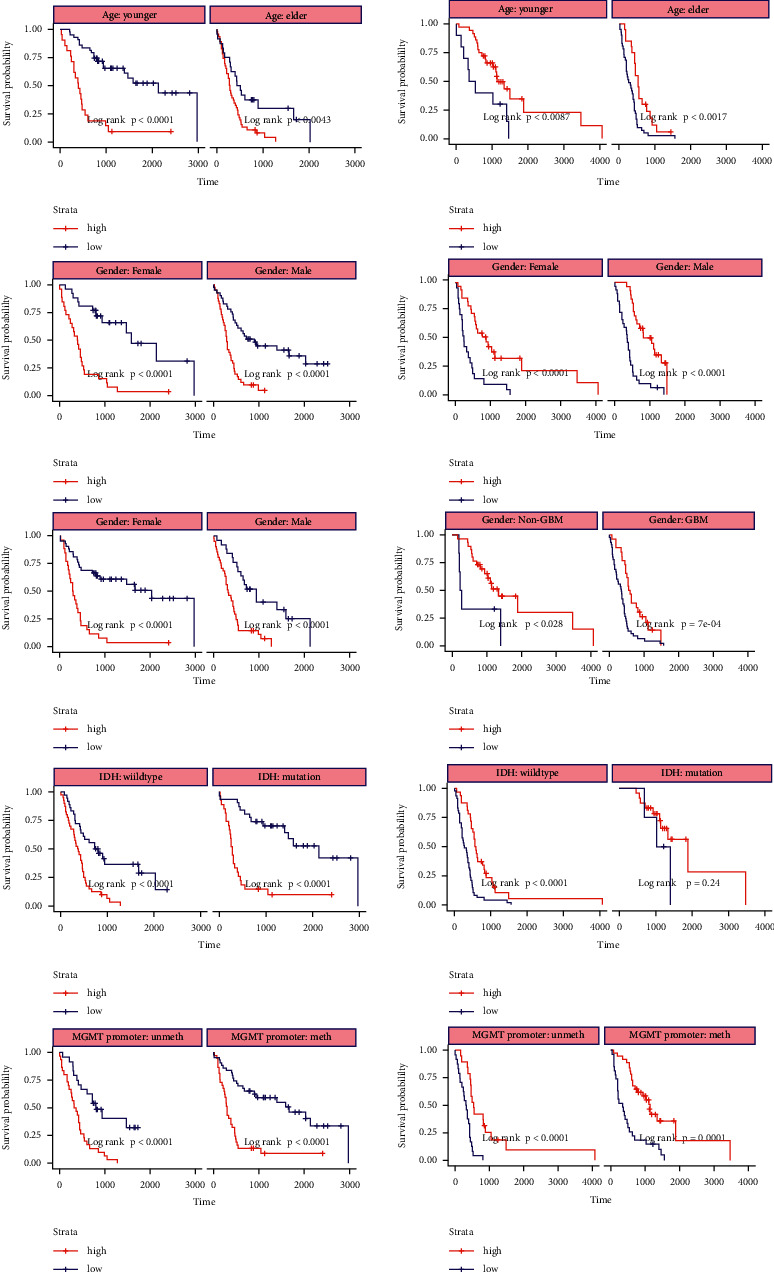
Stratified Kaplan–Meier curves based on different clinical subgroups (younger or older, male or female, and GBM or non-GBM) and molecular subgroups (IDH mutation or wild-type, MGMT promoter methylation or nonmethylation) in (a) training set and (b) validation set.

When the stratified analysis was performed, significant discrimination of the radiomics model between the OS of the high-risk and low-risk groups was observed in different clinical subgroups and molecular subgroups (Figure 7). Based on different clinical (age and grade) and molecular (MGMT promoter methylation status and IDH genotype) risk factors, the radiomics model could further stratify the high-risk and low-risk groups in the training set and external validation set, and significant differences were observed with the log-rank test. Among the subgroups based on gender, tumour grade, and MGMT promoter methylation status, the *p* values in the training set and validation set were all less than 0.0001. In the training set of the age subgroup, the *p* value was less than 0.0001 for the younger group and 0.0043 for the older group. In the validation set of the age subgroup, the *p* value for the younger group was 0.0087, and that for the older group was 0.0017. In the IDH subgroup, the *p* values for the wild-type IDH groups in the training set and the validation set were both less than 0.0001, while for the IDH mutation group in the validation set, the *p* value was 0.24.

### 3.6. Nomogram of the Combined Model

**Figure 8:**
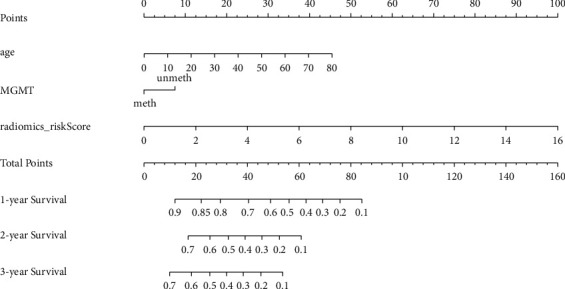
The nomogram integrated age, MGMT promoter state, and radiomics signature to predict 1-year, 2-year, and 3-year OS.

**Figure 9:**
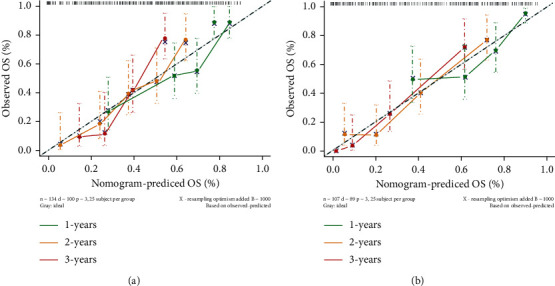
The calibration curves evaluated the agreement between the predicted survival probabilities of the nomogram and actual survival probabilities in the training set (a) and external validation set (b).

**Figure 10:**
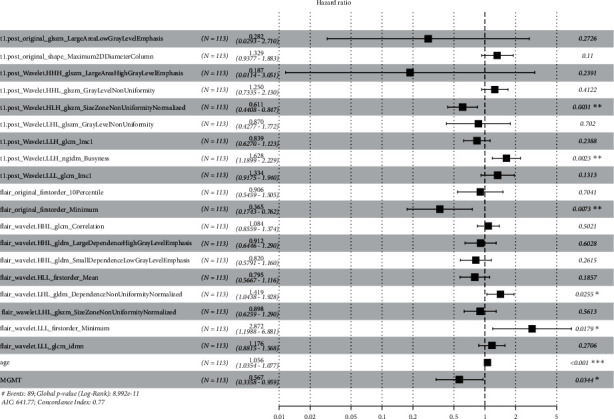
HR values with 95% CIs and *p* values of each feature in the final combined model in the validation set. The C-index of this model is shown on the forest map.

The nomogram incorporating the radiomics signature, MGMT promoter state, and age for OS prediction is illustrated in Figure 8. The Schoenfeld residuals method was used to test the nomogram's proportional hazards assumption, and the results were *χ*^2^ = 26.643 and *p* = 0.183. Calibration curves showed good agreement between the predicted 1-year, 2-year, and 3-year survival probabilities and the actual outcomes for the combined model, especially in the validation set (Figure 9). Compared with the clinical-genetic model (C-index of 0.660 and 0.73 in the training set and validation set, respectively), the radiomics model had higher C-indexes (0.745 and 0.750 in the training set and validation set, respectively). The multivariate Cox model combining the radiomics signature and clinical-genetic factors had the best prognostic prediction performance (C-index of 0.752 and 0.792 in the training set and validation set, respectively) (Figure 10). Furthermore, the integration of the radiomics signature into the clinical-genetic model yielded an NRI of 0.153 (95% CI: 0.009–0.396; *p* < 0.001), which showed improved classification accuracy for survival prediction.

**Figure 11:**
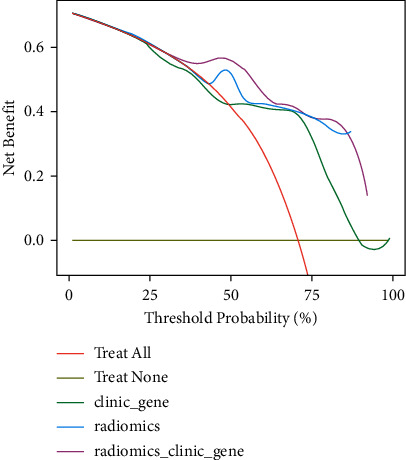
Decision curve analysis for the clinical-genetic model, radiomics model, and combined model. The *y*-axis represents the net benefit, and the *x*-axis represents the threshold probability.

Decision curve analysis showed that if the threshold probability was higher than 25%, the predictive performance was highest for the combined model, followed by the radiomics model and then the clinical-genetic model (Figure 11).

## 4. Discussion

In our study, a comprehensive radiomics analysis integrating MRI features, clinical characteristics and genetic information was performed to preoperatively predict the risk stratification of patients with HGGs. The results showed that the radiomics signature provided significant prognostic value for HGGs, and the combined model integrating the radiomics signature with age and MGMT promoter methylation status achieved the best prognostic predictive performance. Moreover, the incremental prognostic value of the radiomics signature beyond key molecular biomarkers and clinical characteristics was also confirmed through a stratified analysis. Finally, a nomogram incorporating both the clinical-genetic characteristics and radiomics signature was established to individually predict the survival probabilities for patients with HGGs.

HGGs, as the most common primary adult brain malignancy of the CNS, have a poor prognosis [1]. Risk stratification plays an important role in obtaining individual diagnoses and making treatment decisions. Recently, research has increasingly suggested that IDH [4–6] and MGMT [8, 9], which are critical molecular biomarkers, are strong prognostic factors in patients with HGGs; furthermore, MRI radiomics provides another means for the risk stratification of HGGs [23]. A previous study showed that the radiomics signature has better prognostic value than clinical and radiological risk models to predict survival and stratify GBM patients [18], even having incremental value beyond MGMT molecular characteristics [19]. However, some limitations still exist. First, radiomics analysis was only performed on GBMs, and grade III gliomas were not involved; thus, the prognostic value of the radiomics signature in HGGs needs to be investigated. Second, the key prognostic factors (IDH and MGMT) were not compared and integrated in the radiomics analysis, and the incremental prognostic value of the radiomics signature needed further investigation through a comprehensive and stratified risk analysis.

In our comprehensive radiomics analysis, the multivariate Cox model showed that age and MGMT promoter methylation status were the risk factors that should be used to establish a clinical-genetic model. This result may conflict with some previous studies showing that the prognostic value of MGMT promoter methylation states in HGGs depends on the IDH genotype [24]. Radiomics analysis showed that quantitative MRI features extracted from CE-T1 and T2FLAIR MRI images had important prognostic value, and most of these features were texture features and high-dimensional features that represented noise removal and edge enhancement. A radiomics signature combining these features from multiple sequences had an improved predictive performance. This observation emphasizes that using a signature combining different imaging features that describe different aspects of tumour appearance might capture hidden characteristics, offer insight into the heterogeneity of the tumour microenvironment, and thus create a more accurate model to predict the prognosis of patients with HGGs. Our radiomics signature showed superior prognostic value compared to the clinical-genetic risk factors (C-index values of 0.745 vs. 0.660 and 0.750 vs. 0.730 in the training set and external validation set, respectively). Furthermore, this technique has some special advantages, such as its noninvasive, low cost, and real-time prediction, which make it more suitable for clinical practice than the clinical-genetic model based on molecular testing. Despite this, the purpose of our study was not to replace molecular testing but to help radiologists better understand the quantitative MRI features associated with the prognosis of patients with HGGs and identify the most valuable risk factors by comparing the discriminative performances of clinical characteristics, molecular biomarkers, and radiomics signatures.

We also investigated the incremental value of the radiomics signature beyond the clinical characteristics and molecular biomarkers. Stratified Kaplan–Meier survival analysis and log rank tests were performed using the radiomics signature to further identify different risk subgroups among different clinical subgroups (younger or older, male or female, GBM or non-GBM) and molecular subgroups (IDH mutation or wild-type, MGMT promoter methylation or nonmethylation). Significant discrimination between the OS of the high-risk and low-risk groups was observed in both the training set and the external validation set for these subgroups, while the small sample size of the IDH-mutation subgroup in the validation set led to ineffective discrimination of the radiomics signature for risk stratification. Even so, our study demonstrates the incremental value of radiomic features in identifying risk subgroups for patients with HGGs based on different clinical features and molecular biomarkers, although these preliminary results need to be further validated with a larger dataset.

A combined model integrating clinical characteristics, MRI features, and genetic information was also established in our study. The results showed that the combined model incorporating the radiomics signature, age, and MGMT promoter methylation status yielded an excellent prognostic value in both the training set and external validation set compared with the clinical-genetic model and radiomics model alone. This observation emphasizes that incorporating radiomics analysis into clinical practice along with analyses of clinical characteristics and genetic information could further improve the prognostic evaluation of patients with HGGs. Furthermore, to assist clinicians in predicting the survival of patients with HGGs in a more convenient and quantitative manner, a nomogram was established to predict 1-year, 2-year, and 3-year OS probabilities using age, MGMT promoter methylation status, and the radiomics signature. The calibration curves showed good agreement between the predicted and actual survival probabilities in both the training set and the external validation set. This comprehensive radiomics analysis provides a potential tool to guide individual diagnosis and treatment decisions for HGGs.

Although the proposed comprehensive radiomic analysis has some advantages over traditional clinical and molecular risk factors, the limitations of our study merit discussion. First, our model was trained and validated based on different datasets retrospectively collected from the FHSXMU/SPPH and TCGA/TCIA projects, and the heterogeneity of multicentre imaging parameters could not be controlled. Second, our radiomics analysis was only performed on conventional scanning sequences. As our dataset increases in size, more sequences (such as DWI, dynamic susceptibility contrast (DSC) imaging, susceptibility weighted imaging (SWI), diffusion tensor imaging (DTI), and diffusion kurtosis imaging (DKI)) will be included in the radiomics analysis to mine for valuable prognostic information hidden in these MR images to improve the predictive performance.

## 5. Conclusion

In conclusion, our MRI radiomics signature provides a potentially noninvasive biomarker for the risk stratification of patients with HGGs, and it has incremental prognostic value beyond the key molecular biomarkers and clinical characteristics. The combined model integrating clinical characteristics, MRI features, and genetic information has the best predictive performance and may serve as a potential tool to guide individual diagnosis and treatment decisions, although this needs further verification in multicentre and large-scale studies before widespread implementation in clinical practice.

## Figures and Tables

**Table 1:** Patient characteristics in the training set and external validation set.

Characteristic	Training set (*N* = 134)	Ex-validation set (*N* = 107)	*p* value
Age (year)	54 (43–62)	53.93 ± 15.54	0.212
Gender			0.216
Female	52 (38.8%)	50 (46.7%)	
Male	82 (61.2%)	57 (53.3%)	
Histology			0.008
Non-GBM	68 (50.7%)	36 (33.6%)	
GBM	66 (49.3%)	71 (66.4%)	
IDH genotype			0.006
Wild type	76(56.7%)	79 (73.8%)	
Mutation	58(43.3%)	28 (26.2%)	
MGMT promoter			0.986
Unmethylation	54 (40.3%)	43 (40.2%)	
Methylation	80 (59.7%)	64 (59.8%)	
Overall survival (day)	466.5 (253.75–927.25)	508 (254v958)	0.816

*Note.* GBM: glioblastoma; IDH: isocitrate dehydrogenase; MGMT: O6-methylguanine methyltransferase. The training set was from our institution, and the external validation set was from the TCGA/TCIA dataset. A *p* value <0.05 was considered a significant difference.

## Data Availability

The raw data supporting the conclusions of this article will be made available by the authors, without undue reservation.
